# Sickness behaviour and the effect of sex, age, and immune status on individual behavioural variation in *Tenebrio molitor*

**DOI:** 10.1371/journal.pone.0316085

**Published:** 2024-12-31

**Authors:** Antoine Bour, Clint D. Kelly

**Affiliations:** 1 University of Strasbourg, Strasbourg, France; 2 Département des Sciences Biologiques, Université du Québec à Montréal, Montréal, Canada; National Institute of Agricultural Research - INRA, MOROCCO

## Abstract

Sick animals generally behave differently than healthy individuals by, for example, being less active and exploratory. How an individual responds to illness is also likely to be mediated by the individual’s age because age will dictate the individual’s ability to fight a challenge. To date, empirical research on sickness behaviour has focused on the population-level average effect of ill health on behaviour. No study has examined how sickness affects individual behavioural variation, which can affect not only survival and reproductive success but also disease transmission via interactions with conspecifics. In this study, we use a repeated measures design to experimentally test the hypothesis that an immune challenge will induce sickness behaviour in yellow mealworm beetles (*Tenebrio molitor*) and that the effect on behavioural expression will be dose- and age-dependent. We test the prediction that an immune challenge will reduce beetle activity and exploration at the population level as well as modify variation in behavioural expression among individuals with individuals receiving a stronger challenge expressing more sickness behaviour. Although we found little evidence that *T*. *molitor* experiences sickness behaviour, we did find that older beetles were more active than younger ones. There was very little evidence that age, sex, and immune status affect behavioural variation among and within individuals but the phenotypic correlation between activity and exploration is driven by a correlation within individuals. Observed effects within individuals are likely driven by a significant effect of test sequence; behavioural expression significantly decreased in the second of the repeated tests.

## Introduction

An individual animal’s behaviour is often dictated by its state, which is ultimately driven by endogenous (e.g. hormone levels, immune status, body condition, energy reserves, residual reproductive value, metabolic rate) and exogenous (e.g. position in a dominance hierarchy, parasite load, anthropogenic contaminants) factors [1, 2]. One important endogenous factor driving animal behaviour is health. Sick or immune-challenged animals generally behave differently than healthy individuals. For example, sick individuals often adaptively seek shelter while also reducing social interactions, general activity, exploratory behaviour, and food (anorexia) and water (adipsia) consumption [3, 4]. Sickness behaviour is likely adaptive given that remarkably similar behavioural responses are observed across a wide range of animal taxa [reviewed in 5, 6] despite taxa having very different physiologies and immune systems. Indeed, sickness behaviour is hypothesized to conserve energy because these behaviours permit individuals to allocate energy to somatic repair and immunological defences, including fever production [3, 4]. Sickness behaviour is also hypothesized to reduce predation risk by minimizing exposure to predators through reduced activity and increased shelter use [3, 7, 8].

Not all individuals in a population behave similarly when sick, however, because age and sex can dramatically influence how animals adaptively respond behaviourally to an immune challenge. Ageing animals experience senescence, a gradual and irreversible decline in the efficiency of their physiological processes, which includes a deterioration of immune function that can lead to increased pathogen proliferation and increased risk of mortality [9, 10]. Consequently, immunosenescence should cause older immune-challenged individuals to increase their expression of sickness behaviours compared with younger counterparts.

Males and females often respond to disease and pathogens differently, depending on the taxon and type of immune challenge [11, 12], which can manifest as sex differences in sickness behaviour. Unfortunately, most studies of sickness behaviour in animals either pool the sexes [e.g. 5, 13] or use a single-sex experimental design rather than testing both sexes simultaneously [e.g. 14, 15]. Of the few studies that have simultaneously examined sickness behaviour in both sexes, Bouwman and Hawley [16] found that diseased adult male house finches (*Carpodacus mexicanus*) exhibit lower aggression rates than diseased females because sick males are less likely to win an agnostic encounter with a conspecific rival. Ghai et al. [17] found that wild male red colobus monkeys (*Procolobus rufomitratus tephrosceles*) that were infected with a gastrointestinal nematode (*Trichuris* spp.) copulate and rest more and move and groom less, than infected females. Male Sprague–Dawley (SD) rats treated with lipopolysaccharide (LPS) reduce their relative food intake and locomotor activity compared with LPS-treated females [18]. *Gryllus firmus* crickets, on the other hand, do not exhibit any sex differences in sickness behaviour after treatment with LPS [19], and male and female guppies (*Poecilia reticulata*) exposed to fluoxetine do not differ in their activity levels or stress responses [20]. To date, however, no study has explored whether animals exhibit a sex-by-age interaction in their behavioural response to an immune challenge.

The study of state-dependent changes in behaviour has traditionally focused on the (population-level) average response of an individual to endogenous or exogenous variables, with variation around the mean being dismissed as noise [1, 2]. Over the past two decades, however, we have begun to appreciate that this noise, or individual behavioural variation, is not simply the raw material for natural selection but might also be its product. Indeed, behavioural differences among individuals [i.e. behavioural individuality: 2, 21, 22] can be heritable, consistent across time and context, and can strongly influence individual fitness [2, 21, 23–25].

We know that individuals within animal populations vary in their behaviour [2, 21, 22], but less is known about whether individuals respond similarly to the same exogenous and endogenous factors and, if not, why. Differences among individuals in their response to exogenous and endogenous factors are evolutionarily important because they not only expose hidden phenotypic variation to natural and sexual selection, but they can also reveal otherwise hidden life history strategies [2, 26]. For example, differing rates of immunosenescence among individuals likely result in young individuals having similarly strong immune systems while older individuals vary in immune function. Consequently, variation in the expression of sickness behaviour among younger immune-challenged individuals should be smaller than that among older individuals. Otherwise, variation should be similar among individuals if all individuals similarly reduce their behavioural expression when sick regardless of age.

Empirical investigations of sickness behaviour have hitherto concentrated on quantifying the average effects of an immune challenge on behaviour. To our knowledge, no study has experimentally examined how an immune challenge affects behavioural variation among and within individuals and how age and sex mediate that variation. Here, we use a repeated measures design to experimentally test the hypothesis that an immune challenge will induce sickness behaviour in yellow mealworm beetles (*Tenebrio molitor*) and that the effect on behavioural expression will be dose-, sex-, and age-dependent.

**Fig 1:**
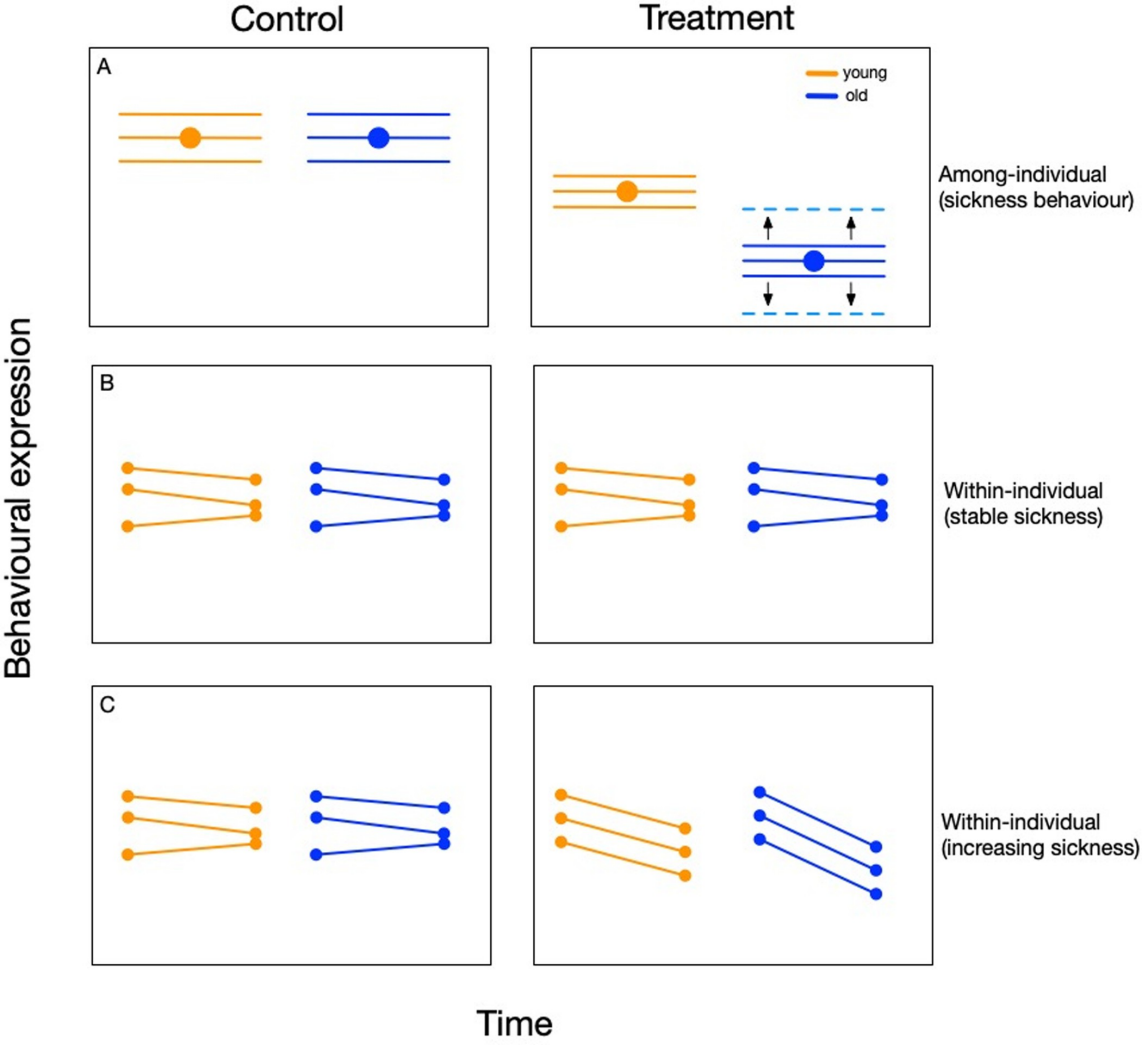
Predicted behavioural expression of young (orange) and old (blue) individuals that do (treatment) or do not (control) receive an immune challenge. (a) If an immune challenge causes sickness behaviour then we expect that young and old immune-challenged individuals will decrease their population-level average behavioural expression compared with controls with old individuals decreasing their behavioural expression more than young individuals. Because immune-challenged individuals are expected to behave similarly, their among-individual behavioural variation should be smaller than that of controls. However, if immune function varies among old individuals due to differing rates of immunosenescence then the variation among old individuals will increase (dashed blue lines). (b) We expect to find little variation in behaviour between behavioural tests in control animals. If an immune-challenged individual’s sickness remains stable (i.e. there is no recovery or increased illness) between behavioural tests then we do not expect to find significant within-individual variation within either age category. (c) However, if there is a change in health between tests (e.g. individuals become sicker) then we expect within-individual variation to increase in old immune-challenged individuals. Solid circles represent the population-level average and horizontal lines represent the behaviour of an individual through time.

We predict that an immune challenge will significantly decrease the population-level average behavioural expression by beetles compared with controls with old immune-challenged individuals having significantly lower behavioural expression (i.e. more sickness behaviour) than young immune-challenged beetles (Fig 1A). We also predict that beetles given a more intense immune challenge will exhibit greater sickness behaviour than otherwise.

How an immune challenge affects among-individual behavioural expression is less clear. One possibility is that immune-challenged beetles will exhibit lower levels of among-individual variation than controls if an immune challenge causes individuals to unanimously reduce their behavioural expression (Fig 1A). Older immune-challenged individuals might more consistently express sickness behaviours than younger immune-challenged individuals if older individuals all have similarly weak immune systems. Alternatively, if the rate of immunosenescence varies among individuals, older individuals could exhibit greater among-individual variation in sickness behaviour (i.e., some will, some will not). Dose-dependent effects on behavioural variation are also unclear. Perhaps a more intense immune challenge will cause individuals to have lower levels of among-individual variation than less intense challenges if an intense challenge causes all individuals to behave even more similarly.

The effect of an immune challenge on within-individual behavioural variation is also difficult to predict because it will depend on how the immune challenge affects individuals between repeated behavioural tests. If individual sickness remains stable between tests then we do not predict any effect of an immune challenge or age on within-individual variation (Fig 1B). However, if individuals become progressively sicker between tests, we predict that within-individual variation will significantly increase in old beetles because of their putatively poorer immune system (Fig 1C).

We explore how an immune challenge affects the sexes at the population and individual levels because we have no *a priori* expectation that females and males will behave differently. By identifying how an immune challenge affects individual behavioural variation in gregarious animals like *T*. *molitor*, our study could provide insight into disease transmission among group members [27].

## Methods

The *T*. *molitor* used in this study were obtained as larvae from Boreal Science (St. Catharines, ON, Canada). We housed the larvae in an environmental chamber set at 25°C, 60% relative humidity, and a 12:12 h light/dark cycle following others [e.g. 28]. Larvae were fed a diet of wheat bran (OnlineOrganics, Quebec, Canada) and fresh organic carrots.

Pupae were sexed [29] and placed in individual plastic 59 mL deli cups (Uline Canada, Milton, ON, Canada). Pupae were checked daily for eclosion to adulthood and the date of eclosion was noted. Adults were provided with a moist cotton ball for water and a medium-quality diet [30] of 75% bran and 25% cellulose. This diet is likely to force individuals to trade off scarce nutritional resources amongst condition-dependent traits and result in greater variance among individuals in fitness-related traits such as immunocompetence whereas a high-quality diet might lead to all individuals maximally investing in all traits. All adults were virgins, and malformed individuals were discarded. Experimental adults were maintained in a Diamed® Percival incubator set at 25°C under a reversed 12:12 LD cycle.

Sexually mature beetles (> 5 days post-eclosion: [31]) were randomly assigned to either a “young” or “old” age category at the time of eclosion to adulthood. Young beetles were treated experimentally 9 days after eclosion, whereas old beetles were treated 31 days after eclosion. The age of old beetles was a compromise between the life expectancy of adults and the logistic time constraints of the study [28].

Beetles were randomly assigned to one of five experimental treatments. Beetles either (i) received no treatment apart from handling; (ii) were injured by puncturing the pleural membrane as a wounding control; (iii) were injected with 5 *μ*l sterile phosphate-buffered saline (PBS, Sigma-Aldrich, St. Louis, MO, USA) as an injection control; (iv) were injected with 0.5 mg of lipopolysaccharide derived from the gram-negative bacterium *Serratia marcescens* (LPS, Sigma-Aldrich, St. Louis, MO, USA) that was suspended in 5 *μ*l of PBS (low dose); or (v) injected with 1.0 mg of LPS suspended in 5 *μ*l of PBS (high dose). LPS is a non-pathogenic and non-living elicitor that stimulates several pathways in the insect immune system [32–34], including that of *T*. *molitor* [35]. We applied two different concentrations of LPS to beetles to explore whether their behavioural response to an immune challenge is dose-dependent. Our low dose was the same as that administered to *T*. *molitor* by Haine et al. [35], which had a significant immunological effect for several days. Our high dose was twice that of the low dose. All injections and wounding were given into the haemocoel between the third and fourth abdominal sternites using a 10-mL Hamilton syringe equipped with a 26s-gauge needle [35, 36]. Beetles were injected under a Leica® S6D stereomicroscope (Leica Microsystems Inc., Concord, ON, Canada). All utensils, needles, and surfaces were wiped or rinsed with 95% ethanol in between injections to remove cuticular compounds. Separate syringes were used for each treatment. Treated beetles were placed in a deli cup without food to avoid them acquiring nutritional resources to fuel an immune response. Only water was provided in the form of a moistened cotton ball. We measured the body mass of each beetle before treatment and after the second behavioural test to the nearest 0.0001 g by using a Sartorius Secura 224-1S analytical balance.

Each beetle’s behaviour was recorded twice with the first test occurring approximately 38 ± 2 hours after treatment and the second approximately 62 ± 2 hours after treatment (i.e. 24 h after the first test). Our post-treatment observation times fall within the window of *T*. *molitor*’s peak immune response to LPS [35]. *T*. *molitor* is most active at night because it is a nocturnal species [37]. We therefore conducted our behavioural tests between 0900 and 1000 hours (i.e. 2–3 hours after lights out), which is the beginning of their scotophase in the lab.

Test arenas were plastic Petri dishes (9 cm diameter) lined with filter paper for traction. At the time of the trial, beetles were taken from their deli cup and placed in the centre of a Petri dish by using broad-tip entomological forceps. Petri dishes were placed on top of an infrared backlight array (Noldus Information Technology Inc.) and under a Basler Ace monochrome IR-sensitive camera with Gigabit Ethernet interface. Beetle behaviour was recorded by using Ethovision® XT tracking software [38]. Each beetle’s movement was tracked in the dark for 10 minutes with recording beginning once the beetle was detected in the arena by the software. The Petri dishes were cleaned with 95% ethanol and the filter paper was replaced between tests to prevent cuticular compounds from influencing behaviour [36]. After an individual’s second test, they were weighed for a second time and then euthanized by being placed in a -20˚C freezer. Individuals were stored frozen until later dissection to verify sex determination.

We quantified two behaviours using EthoVision. First, we measured the total distance travelled (cm) by each beetle in 10 minutes. Second, we measured the latency (s) to visit each quadrant of the Petri dish at least once. A beetle could visit all quadrants quickly if they moved in a tight circle in the middle of the arena where all quadrants meet. To avoid this scenario, we created a 4.5 cm diameter hidden zone in the arena’s centre; animals are not tracked by Ethovision in hidden zones. This ensured that latency was recorded for beetles visiting the exterior 2.25 cm portion of each quadrant.

### Ethical note

Our research followed the ASAB/ABS Guidelines for the use of animals in research. *Tenebrio molitor* is not listed as a threatened species and requires no licence to be studied in Canada. Beetles were maintained under optimal laboratory conditions and were freeze-killed prior to dissection.

### Statistical analysis

We removed individuals from the final dataset that died during the study (n = 14) or that escaped during testing (n = 2). Our final sample sizes (and summary statistics) are given in Table 1. Statistical analyses were performed within the R v. 4.2.1 statistical environment [39]. We assessed the impact of our treatments on body mass by using an analysis of covariance (ANCOVA) wherein body mass after treatment was entered as the response variable and initial body mass was entered as a covariate. ANCOVA is the preferred method for analyzing pre-post data rather than ANOVA on change scores or repeated measures ANOVA, which can be biased when regression toward the mean is present [40, 41]. Sex, age, and immune treatment were also entered into the model as fixed factors.

**Table 1:** Mean (± SD) activity (distance travelled, cm) and exploration (latency to visit all quadrants of arena, s) for young (9-d old) and old (31-d old) adult female and male *Tenebrio molitor* beetles that received one of five experimental treatments. Means for each individual (N) were used to calculate group means. Two repeated behavioural measures (separated by 62 ± 2 h) were taken for each of the N = 929 beetles giving N = 1858 behavioural observations.

	Young			Old		
Treatment	Travel distance (cm)	Quadrant visitation (s)	N	Travel distance (cm)	Quadrant visitation (s)	N
***(a) Females***						
Handled	347.8 ± 176.3	546.4 ± 77.6	48	373.3 ± 200.3	514.5 ± 110.6	47
Injured	378.7 ± 226.1	553.3 ± 52.7	48	431.4 ± 262.0	538.4 ± 97.0	45
Saline	387.5 ± 199.5	551.5 ± 71.2	45	392.3 ± 206.0	525.4 ± 106.6	49
Low LPS	322.6 ± 179.6	543.4 ± 63.1	46	419.5 ± 230.9	526.0 ± 90.5	44
High LPS	353.0 ± 211.8	536.1 ± 89.7	46	382.1 ± 212.2	510.3 ± 104.8	50
***(b) Males***						
Handled	356.7 ± 172.7	550.9 ± 50.4	49	402.8 ± 228.1	521.6 ± 109.3	49
Injured	337.6 ± 193.8	535.2 ± 76.7	44	380.1 ± 192.6	527.6 ± 88.4	48
Saline	317.6 ± 182.7	528.4 ± 81.4	48	443.6 ± 249.4	548.3 ± 60.3	46
Low LPS	344.2 ± 168.7	531.0 ± 107.3	43	457.1 ± 215.6	537.4 ± 83.3	45
High LPS	357.4 ± 226.1	523.9 ± 98.0	44	426.4 ± 225.7	539.4 ± 66.6	48

The variable total distance travelled (cm) was Yeo-Johnson transformed to approximate a Gaussian error distribution [42]. We adjusted the latency to visit all quadrant scores by subtracting an individual’s time from the maximum time available (600 s) so that higher values represent more exploratory individuals. This variable was orderNorm transformed to approximate a Gaussian error distribution [43]. All data transformations were performed using the R package *bestNormalize* [44].

We report posterior means with 95% credibility intervals (CrI) from Bayesian generalized linear mixed-effects models [*brms* package: 45]. Inference was based on CrIs that did not overlap zero. Models were run for 8000 iterations (500 warmups) on 4 chains, using relatively uninformative, default priors and a thinning interval of 2 (total post-warmup samples = 9000). Posterior predictive checks were performed to ensure adequate model fits and trace plots confirmed that models converged with low among-chain variability (Rhat = 1.00).

We investigated the average effects of treatment, age, and sex on travel distance and latency to visit all quadrants using multivariable general linear mixed-effects models. We constructed two models for each of our two dependent behavioural variables and compared them using leave-one-out cross-validation (LOO) [46]. First, we built a full model having age (two levels), sex (two levels), treatment (five levels), test number (two levels) and their interactions as fixed effects (model 1). We then removed the interactions if non-significant and re-ran the model (model 2). In all models, individual ID was included as a random intercept separately for each age, sex and treatment combination. An age-by-sex-by-treatment interaction in the residual part of the models was included to estimate the within-individual (residual) variance for each combination of age, sex, and treatment. This model structure permitted us to test not only for the effects of age, sex, and treatment on average behaviours but to also quantify their effects on among-individual (*Δ*V_A_) and residual within-individual behavioural variance (*Δ*V_W_) separately for each age, sex, and treatment combination [see 20, 47]. Both dependent variables were scaled (mean = 0, SD = 1) before analysis to aid model fitting and we assumed Gaussian error structure for both variables. Test number was left-centered (i.e., test 1 = 0) to set the model intercept at the first trial. Inclusion of the covariate body mass was not statistically significant and was removed from the models.

We calculated magnitude differences (*Δ*V) in both among- (*Δ*V_A_) and within- (*Δ*V_W_) individual variances to test whether these components of variation differed between treatments in young and old female and male beetles [48]. We leveraged our Bayesian framework to directly estimate the distribution of *Δ*V by taking the difference in the posterior distribution of each combination of interest. We interpret the posterior mode of ΔV as the estimated strength of ΔV, with 95% credible intervals representing the precision around our estimates [48].

We calculated the unpartitioned phenotypic correlation between distance travelled and latency to visit all quadrants for each age, sex, and treatment combination using Pearson’s product-moment correlation (*r*_p_ ± 95% confidence intervals). We then partitioned the correlations into their components among (*r*_ind_) and within (*r*_e_) individuals by running separate bivariate linear mixed-effects models for each age, sex, and treatment combination. Each model included distance travelled and latency to visit all quadrants as response variables and individual ID as a random intercept. Like the univariate models above, we ran the bivariate models on four chains for 8000 iterations (1000 warmups), using weakly informative, default priors.

All data were visualized using the R packages *ggplot2* [49] and *tidybayes* [50].

## Results

Beetles (n = 929) were equally allocated to each age (F = 1.18, df = 1, 909, p = 0.279) and treatment (F = 1.57, df = 1, 909, p = 0.180) category according to body mass. Injured (Estimate = -3.33 ± 0.98, t = -3.41, p<0.001), young (Estimate = -2.24 ± 0.97, t = -2.30, p = 0.022), and male (Estimate = -3.02 ± 0.96, t = -3.14, p = 0.002) beetles lost significantly more body mass throughout the experiment than their counterparts.

### Population-level sources of variation in behaviour

Reduced models without interactions were the best fit (S1 Table in S1 File). We found little evidence that sex affects distance travelled or latency to visit all quadrants in *T*. *molitor* (S2, S3 Tables in S1 File). Old beetles travelled significantly farther than young ones [Estimate = -0.17 (-0.28, -0.07); S2 Table in S1 File] but age did not have a significant effect on the latency to visit all quadrants [Estimate = 0.03 (-0.07, 0.14); S3 Table in S1 File]. We found strong evidence that test sequence strongly affects behaviour as beetles travelled farther [Estimate = -0.43 (-0.50, -0.35); S2 Table in S1 File] but required more time to visit all quadrants [Estimate = -0.31 (-0.38, -0.23); S3 Table in S1 File] in the first test compared to the second (S2 and S3 Tables in S1 File).

### Among- and within-individual behavioural variation

**Fig 2:**
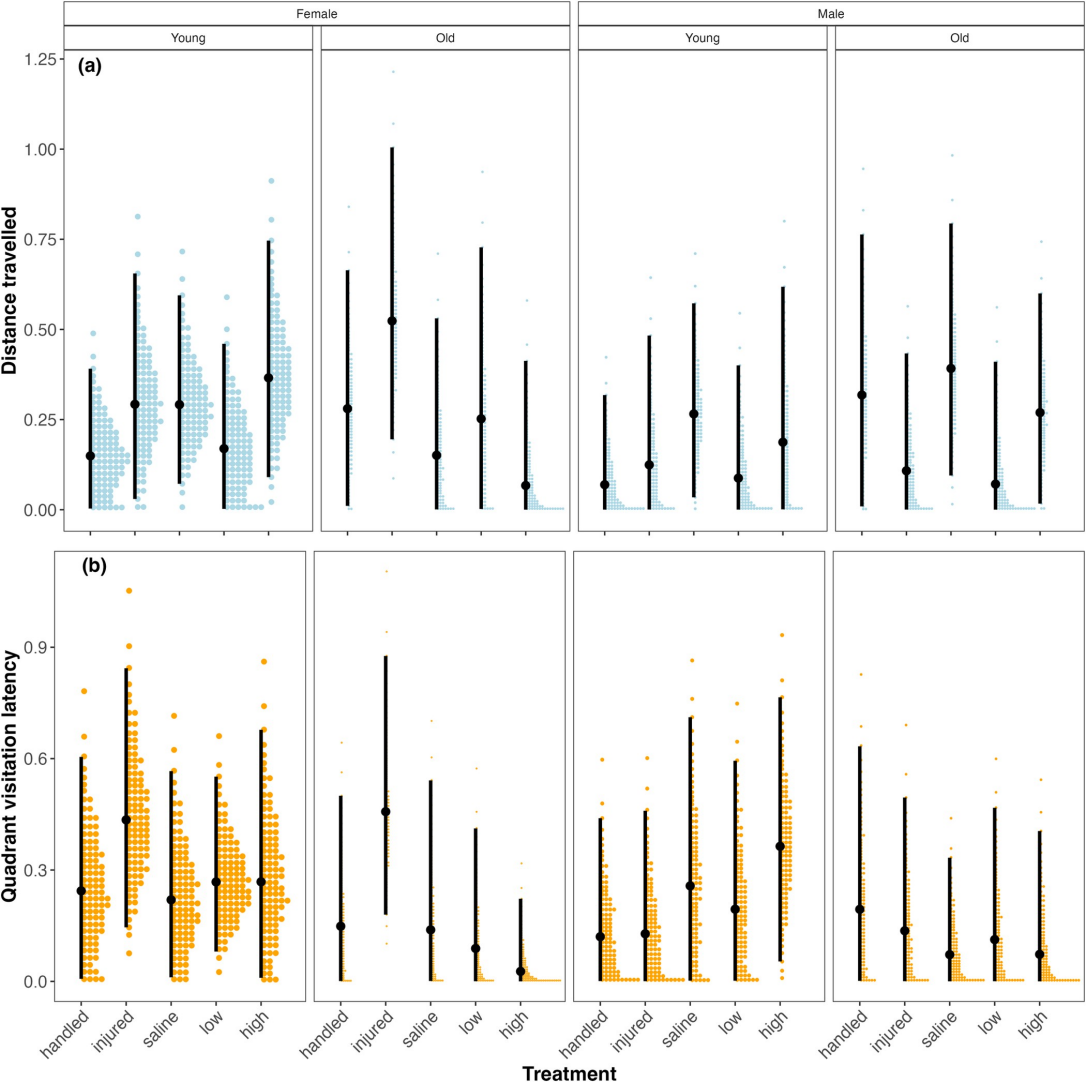
Among-individual variation in distance travelled and latency to visit all quadrants for each treatment, age, and sex combination. See Table 1 for sample sizes.

**Fig 3:**
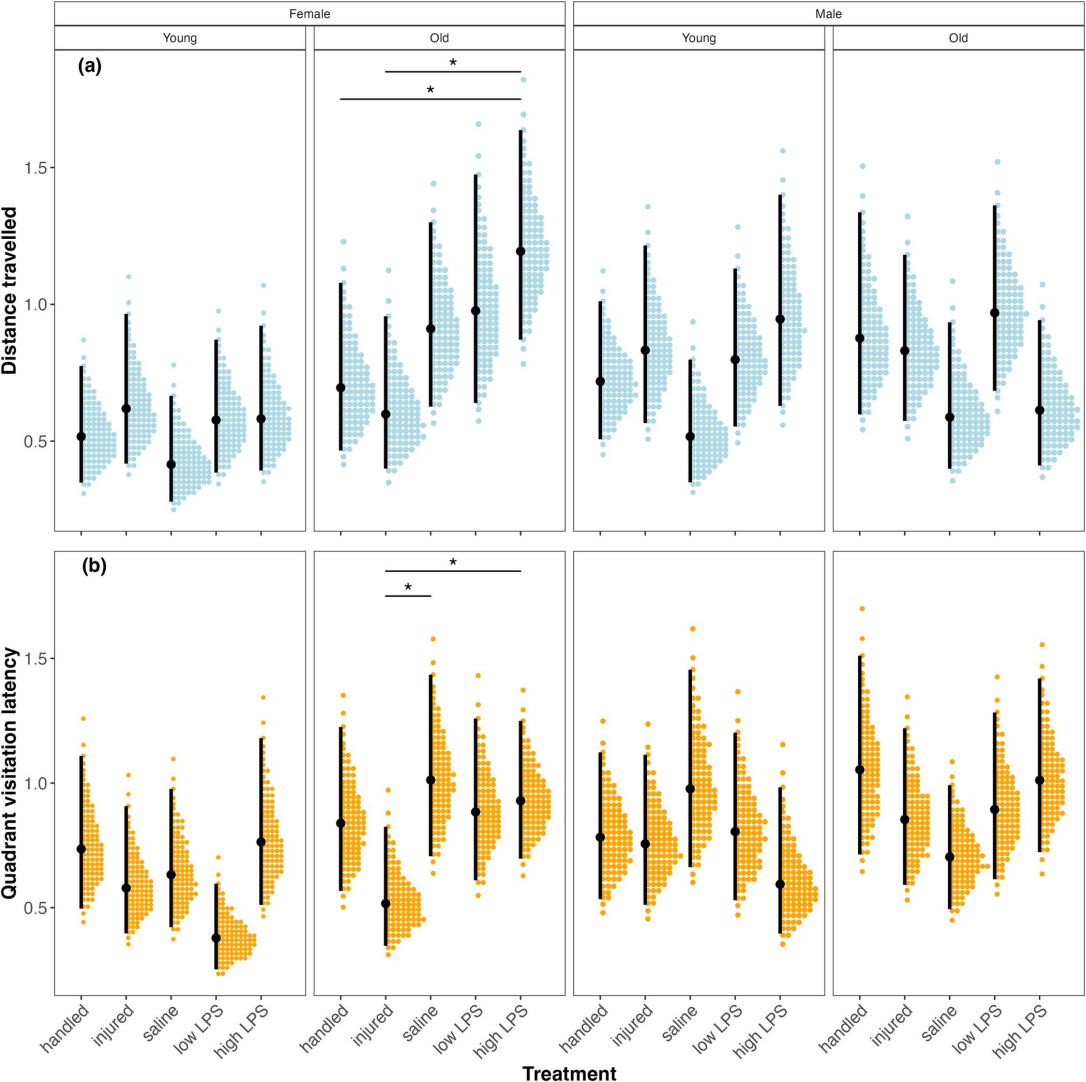
Within-individual variation in distance travelled and latency to visit all quadrants for each treatment, age, and sex combination. See Table 1 for sample sizes. Asterisks denote that the 95% CrI of treatment contrasts do not overlap zero.

We found little evidence that our immune challenges affected the among-individual behavioural variation for any combination of sex and age (Fig 2 and S4 Table in S1 File). In contrast, we found evidence that treatment with a high dose of LPS affects within-individual variation in old females. Specifically, old females treated with a high dose of LPS exhibited greater within-individual variation in travel distance than either handled or saline-injected controls (Fig 3 and S5 Table in S1 File). Old females that were injured exhibited significantly less within-individual variation than old females injected with either a high dose of LPS or saline (Fig 3; S5 Table in S1 File).

### Bivariate correlations

**Fig 4:**
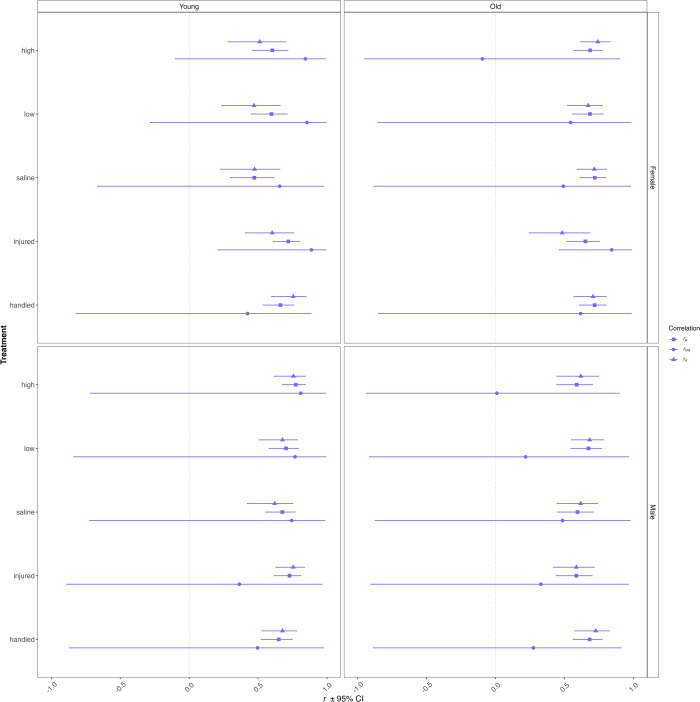
Estimates for unpartitioned phenotypic (r_p_), among-individual (r_ind_), and within-individual (r_e_) correlations between distance travelled and latency to visit all quadrants for each treatment, age, and sex combination (N = 929 beetles). Uncertainty represented by 95% confidence intervals for unpartitioned estimates and 95% credible intervals for among- and within-individual estimates.

We found strong evidence that the unpartitioned phenotypic correlation (Pearson product-moment correlation, *r*_p_) between distance travelled and latency to visit all quadrants is positive in all cases (Fig 4). Among-individual correlations (*r*_ind_) were significant in only two of 20 cases with both being for young and old females that were injured. Within-individual correlations (*r*_e_) were significant in all 20 cases.

## Discussion

Considerably more empirical effort has been allocated to quantifying the long-lasting effects of an immune challenge on personality-related behavioural traits [e.g. 51, 52, 53] than on their transient effects (e.g. sickness behaviour) within a single life stage (e.g. adulthood). Studies that have examined the relationship between immunity and personality in adults have typically done so by measuring the immune response of individuals differing in some measure of personality [e.g. 54, 55, 56, 57]. Fewer studies, however, have investigated the effect of an immune challenge on individual behavioural variation, as we have done here. In this study, we immune-challenged adult female and male *T*. *molitor* beetles to test the hypothesis that because immune activation elicits sickness behaviour, beetles will adaptively reduce their activity to conserve energy for the immune response. This behavioural change should be revealed as a decline in the population-average behaviour and a possible reduction in its variation. Contrary to prediction, we found no interactive effects of sex, age, or immune treatment on the population-level average behaviour of *T*. *molitor* beetles, as measured by the total distance travelled and the average latency to visit all of the arena’s quadrants. Neither did we find significant effects of our fixed factors on among- or within-individual behavioural variation. We did, however, find that the average distance travelled was dependent on relative beetle age irrespective of their sex or immune status. Following other studies of individual behavioural variation [22], we interpret distance travelled in an open field as a measure of activity and the latency to visit all quadrants as a measure of exploration. These are generally deemed fitness-relevant traits since they are associated with the ability to disperse [58–60] and find mates [e.g. 61] and also fall within the repertoire of sickness behaviour [4].

At the population level, our immune-challenged beetles did not express reduced activity or exploration, two characteristics of sickness behaviour. We expected that an immune challenge, particularly a high dose, would reduce the activity and exploration of our beetles but it did not. Kelly and McCabe Leroux [19] and Sullivan et al. [5] similarly found no effect of an immune challenge on activity in *Gryllus firmus* and *G*. *texensis* crickets, respectively. Most studies, however, find that animals reduce behavioural activity after immune challenge [3, 4]. For example, pigs are less active for several hours following LPS treatment [62] and *Octopus vulgaris* octopi exhibit lower levels of general activity after receiving an injection of LPS [63]. That an immune challenge did not significantly affect average behavioural responses was not due to challenged beetles fueling their immune response by increasing their consumption of food since beetles were not fed post-treatment. It is also possible that LPS is ineffective at stimulating an immune response, and thus sickness behaviour, in *T*. *molitor*; however, Haine et al. [35] showed that LPS stimulates antimicrobial activity in *T*. *molitor* hemolymph for several days post-administration. We administered doses equal to and double those administered by Haine et al. [35] without any effect on behaviour. However, we might have observed a stronger effect of our LPS treatment on beetle behaviour if we had used LPS derived from *Escherichia coli* rather than *S*. *marcescens* [35, 64]. Differential fitness effects of *E*. *coli* and *S*. *marcescens* have been reported in insects [e.g. 64]. Another possibility is that *T*. *molitor* do not express sickness behaviour through reduced activity but rather through increased anorexia and adipisia. Perhaps our LPS-treated beetles did not modify their behaviour because they lack physiological fever [65, 66]. Infected mammals, for example, are expected to conserve their energy for physiological fever by reducing their activity [8] whereas invertebrates will move to warmer areas to raise their body temperature and thus might not require greater quiescence.

We found that age had a significant effect on beetle activity at the population level with old beetles being more active than young individuals. That young beetles were less active is counter-intuitive given that senescence is expected to slow individuals as they age. For example, locomotion and activity become increasingly impaired as fruit flies (e.g. *Drosophila* spp.) and cockroaches (e.g. *Blaberus* spp.) age [67, 68]. In contrast, Rodríguez-Muñoz et al. [69] found that the mate-search activity of male *G*. *campestris* field crickets in the wild did not diminish over their lifespan and Wilson et al. [70] found that age explained little of the variation in activity observed in *Acheta domesticus* house crickets.

Older individuals in our study might have been more active than younger beetles because they were terminally investing in reproduction [sensu 71]. If older individuals perceived their residual reproductive value to be low, then they might have adaptively increased their investment in mating-related behaviours. This hypothesis assumes that our measure of activity is integral to *T*. *molitor* reproduction by, for example, representing investment in mate searching. Although older adult beetles being more active supports the terminal investment hypothesis, this hypothesis predicts that immune-challenged individuals will exhibit higher levels of behavioural expression than controls because their residual reproductive values should be lowest [72]. This was not the case. Perhaps age is the primary factor influencing terminal investment in *T*. *molitor* and it simply overwhelms any other contributing effects. Rutkowski et al. [73] similarly showed that age had a stronger effect on terminal investment than immune status in male *Teleogryllus oceanicus* as old males produced larger spermatophores than young males whereas immune status had no significant effect on spermatophore size. It is also possible that old beetles in our study were in better body condition than young ones because they had more time to acquire nutritional resources, and could thus invest more in mating-related behaviours than young beetles [e.g. 74].

Sex did not significantly affect beetle activity or exploration. This finding contrasts with studies showing that female animals often move more than males [75] and vice versa [5, 61, 76]. Differences between the sexes in mobility are generally driven by sexual selection with the mate-seeking sex, usually males, travelling farther distances than the opposite sex, usually females [77]. Our findings suggest that sexual selection might not operate on locomotion or mobility in *T*. *molitor* or it might be equally strong in both sexes.

We expected immune-challenged individuals to universally exhibit sickness behaviour compared to controls, meaning all beetles would similarly reduce their activity and exploration. This behavioural shift was predicted to result in significantly smaller among-individual variation in immune-challenged individuals. We alternatively predicted that among-individual variation might significantly increase in old beetles if the rate of immunosenescence varies among individuals, with some individuals expressing more intense sickness behaviour than others. Contrary to prediction, none of our treatment conditions significantly affected the among-individual variation observed in beetle activity or exploration. We found that beetles neither increased nor decreased their behavioural variation in response to an immune challenge or their sex or relative age; beetles behaved similarly regardless of intrinsic or extrinsic factors. Again, we might not have observed any treatment effects on behaviour because, for example, *T*. *molitor* does not exhibit sickness behaviour, LPS derived from *S*. *marcescens* does not stimulate its immune system [e.g. 64] to the degree that *E*. *coli* does [e.g. 35], or our old (31-d) beetles were not immunosenescent [78].

Within-individual variation describes the consistency of individuals in repeated expressions of behaviour. We know of no studies investigating the effect of an immune challenge on within-individual behavioural variation. However, studies on a variety of taxa (e.g. crabs: [79]; snails: [80]; fish: [20]) have found that exposure to environmental pollutants reduces within-individual behavioural variation (i.e. made individuals less behaviourally plastic). Although we found no general effect of our treatment conditions on within-individual behavioural variation in *T*. *molitor* we did find that in two cases within-individual behavioural variation was significantly affected by immune treatment in old females. First, within-individual variation in activity was significantly higher in old females given a high dose of LPS than in old females that were either handled or injured. Second, we found that old females that were injured expressed less within-individual variation in exploration than old females that were injected with either a high dose of LPS or saline. These results could arise from some individuals increasing or decreasing (or both) their activity and exploration in the second test. We suggest that it was likely due to a general decrease in activity in the second test given that beetles significantly decreased their population-level average activity and exploration the second time they were tested. Our finding raises the question of why beetles would exhibit less activity and exploration in subsequent tests.

Our beetles might have invested less in activity and exploration in the second test because they were sicker in this test than in the first. This possibility, however, seems unlikely as control beetles exhibited the same pattern (i.e. there was no test sequence x treatment interaction) suggesting that it was generally unrelated to individual health status. Alternatively, the observed behavioural decrement suggests that beetles might have habituated to the test [81]. Habituation is a simple form of learning that arises from repeated stimulation and has been demonstrated in a wide variety of taxa including snakes [e.g. 82], fish [e.g. 83], crabs [e.g. 84], mammals [e.g. 85], lizards [e.g. 86], birds [e.g. 87], and even in insects [e.g. 88]. Most demonstrations of habituation result from repeated observations over a relatively short interval. For example, adult male wall lizards (*Podarcis muralis*) habituated to simulated predator-attacks that were repeated every 10 minutes for 1.5 h [86], or penguins habituated to daily disturbances over several days [e.g. 89], and *T*. *molitor* larvae habituate to stimuli presented constantly over 13 minutes [88]. Therefore, it seems unlikely that our beetles became habituated to either our procedures or the test arena given that they were exposed to it only once for 10 minutes 24 h before.

We found, as expected, that phenotypic correlations between activity and exploration were significantly positive within each sex, age, and treatment combination. Monceau et al. [57] similarly found that activity and exploration were positively correlated in adult *T*. *molitor* and studies on other taxa demonstrate a positive association between activity and exploration [59, 90, 91]; however, others have not [70].

Before concluding that travel distance (activity) and quadrant visitation (exploration) comprise a behavioural syndrome, it is necessary to show that an among-individual correlation is responsible for the phenotypic correlation. For example, phenotypic correlations between aggression and boldness in Belding’s ground squirrels (*Urocitellus beldingi*) [90] and between risk-taking behaviours in Wellington tree weta (*Hemideina crassidens*) [92] appear to be driven by among-individual correlations; that is, they are due to a relationship between individuals’ average levels of two behaviours. Partitioning our phenotypic correlations revealed that our phenotypic correlations are generally driven by variation within, rather than among, individuals. In other words, individuals similarly increased or decreased their behaviours between the two tests. The lack of among-individual correlations thus erodes our confidence that the observed phenotypic association between activity and exploration comprises a behavioural syndrome [93]. This is not surprising given that beetles in our study were significantly more active and exploratory in their first test than their second one. That within-individual correlations are driving phenotypic correlations is in line with Bell & Stamps [94] who similarly documented a positive phenotypic correlation between boldness and aggression in three-spined sticklebacks (*Gasterosteus aculeatus*), but found that individuals with low boldness and aggression on one occasion were just as likely to have high boldness and aggression on the next. Moreover, the negative phenotypic correlation between the maximum distance from the burrow and the time to emerge after disturbance in *G*. *campestris* field crickets is also driven by a correlation at the within-individual level [95].

In conclusion, we found little evidence that *T*. *molitor* experiences sickness behaviour. However, our work reveals that older beetles were more active than younger ones. There was very little evidence that age, sex, or immune status affect behavioural variation among and within individuals. A correlation within individuals drives the phenotypic correlation between activity and exploration at every factorial combination. The within-individual effects observed in our study are likely driven by a significant effect of test sequence on beetle behaviour given that the behaviour of our test subjects significantly decreased in the second of two tests.

## Supporting information

S1 File (PDF)
