# Supplementary material for: Sickness behaviour and the effect of sex, age, and immune status on individual behavioural variation in Tenebrio molitor
Source: PLoS One. 2024 Dec 31;19(12):e0316085. doi: 10.1371/journal.pone.0316085 (PMC11687652; doi:10.1371/journal.pone.0316085)
Supplement: S1 File — (PDF) [file pone.0316085.s001.pdf]

Supplemental Material: Sickness behaviour and the effect of sex, age, and immune status on individual behavioural variation in *Tenebrio molitor*

Clint D. Kelly[1,✉], and Antoine Bour[1]

[1] Département des Sciences biologiques, Université du Québec à Montréal, Montréal, Canada

✉ Correspondence: Clint D. Kelly <kelly.clint@uqam.ca>

Table S 1: Comparison of full (with interactions) and reduced (no interactions) Bayesian linear mixed-effects models for each of three behaviours using difference in expected log predictive density (elpd). Models have either all three control treatments (handled, injured, or manipulated) as separate factor levels or pooled (single control factor-level).

**Model**	**elpd difference ± SE**
**(a) Activity (total distance travelled, cm)**	
(2) activity ~ sex + treatment + age + assay + (0 + sex:age||gr(id, by = treatment)), sigma ~ 0 + age:sex:treatment	0 ± 0
(1) activity ~ sex * treatment * age + assay + (0 + sex:age||gr(id, by = treatment)), sigma ~ 0 + age:sex:treatment	-13.9 ± 6.2
**(b) Exploration (latency to visit all quadrants, s)**	
(2) exploration ~ sex + treatment + age + assay + (0 + sex:age||gr(id, by = treatment)), sigma ~ 0 + age:sex:treatment	0 ± 0
(1) exploration ~ sex * treatment * age + assay + (0 + sex:age||gr(id, by = treatment)), sigma ~ 0 + age:sex:treatment	-7.7 ± 3.3

Table S 2: Estimates [± 95% credible intervals] from Bayesian linear mixed-effects models investigating the effect of immune challenge by lipopolysaccharide (LPS) on activity (distance travelled, cm) of young and old female and male Tenebrio molitor beetles. Fixed effect estimates that differ from zero are in bold text. Female (sex), old (age), and high LPS (treatment) are the fixed effects reference levels. Random effects are presented in standard deviation (SD) units and random variances in log SD units.

	**Estimate [95% CI]**
Fixed effects	
Intercept	0.28 [0.14, 0.42]
sexMale	0.00 [-0.10, 0.10]
treatmentlow	0.03 [-0.13, 0.19]
treatmentsaline	0.04 [-0.12, 0.21]
treatmentinjured	0.02 [-0.15, 0.18]
treatmenthandled	0.01 [-0.14, 0.17]
**ageYoung**	**-0.18 [-0.28, -0.07]**
**assay2**	**-0.43 [-0.50, -0.35]**
Residual variances	
ageOld:sexFemale:treatmenthigh	0.09 [-0.07, 0.25]
ageYoung:sexFemale:treatmenthigh	-0.27 [-0.47, -0.04]
ageOld:sexMale:treatmenthigh	-0.24 [-0.44, -0.03]
ageYoung:sexMale:treatmenthigh	-0.03 [-0.23, 0.17]
ageOld:sexFemale:treatmentlow	-0.01 [-0.22, 0.19]
ageYoung:sexFemale:treatmentlow	-0.27 [-0.48, -0.07]
ageOld:sexMale:treatmentlow	-0.02 [-0.19, 0.15]
ageYoung:sexMale:treatmentlow	-0.11 [-0.30, 0.06]
ageOld:sexFemale:treatmentsaline	-0.05 [-0.23, 0.13]
ageYoung:sexFemale:treatmentsaline	-0.44 [-0.64, -0.20]
Random intercepts	
ageOld:sexMale:treatmentsaline	-0.27 [-0.46, -0.03]
ageYoung:sexMale:treatmentsaline	-0.33 [-0.53, -0.11]
ageOld:sexFemale:treatmentinjured	-0.26 [-0.46, -0.02]
ageYoung:sexFemale:treatmentinjured	-0.24 [-0.44, -0.02]
ageOld:sexMale:treatmentinjured	-0.09 [-0.28, 0.08]
ageYoung:sexMale:treatmentinjured	-0.09 [-0.28, 0.10]
ageOld:sexFemale:treatmenthandled	-0.18 [-0.38, 0.04]
ageYoung:sexFemale:treatmenthandled	-0.33 [-0.53, -0.13]
ageOld:sexMale:treatmenthandled	-0.07 [-0.26, 0.14]
ageYoung:sexMale:treatmenthandled	-0.17 [-0.34, 0.01]
sexFemale:ageOld:treatmenthigh	0.26 [0.01, 0.64]
sexMale:ageOld:treatmenthigh	0.52 [0.13, 0.77]
sexFemale:ageYoung:treatmenthigh	0.60 [0.30, 0.86]
sexMale:ageYoung:treatmenthigh	0.43 [0.03, 0.79]
sexFemale:ageOld:treatmentlow	0.50 [0.04, 0.85]
sexMale:ageOld:treatmentlow	0.27 [0.01, 0.64]
sexFemale:ageYoung:treatmentlow	0.41 [0.05, 0.68]
sexMale:ageYoung:treatmentlow	0.30 [0.02, 0.63]
sexFemale:ageOld:treatmentsaline	0.39 [0.03, 0.73]
sexMale:ageOld:treatmentsaline	0.63 [0.31, 0.89]

sexFemale:ageYoung:treatmentsaline	0.54 [0.27, 0.77]
sexMale:ageYoung:treatmentsaline	0.52 [0.19, 0.76]
sexFemale:ageOld:treatmentinjured	0.72 [0.44, 1.00]
sexMale:ageOld:treatmentinjured	0.33 [0.02, 0.66]
sexFemale:ageYoung:treatmentinjured	0.54 [0.17, 0.81]
sexMale:ageYoung:treatmentinjured	0.35 [0.02, 0.69]
sexFemale:ageOld:treatmenthandled	0.53 [0.10, 0.81]
sexMale:ageOld:treatmenthandled	0.56 [0.10, 0.87]
sexFemale:ageYoung:treatmenthandled	0.39 [0.06, 0.63]
sexMale:ageYoung:treatmenthandled	0.26 [0.02, 0.56]
Num.Obs.	1864
R2	0.275
RMSE	0.78

Table S 3: Estimates [± 95% credible intervals] from Bayesian linear mixed-effects models investigating the effect of immune challenge by lipopolysaccharide (LPS) on exploration (time to visit all quadrants) of young and old female and male Tenebrio molitor beetles. Fixed effect estimates that differ from zero are in bold text. Female (sex), old (age), and high LPS (treatment) are the fixed effects reference levels. Random effects are presented in standard deviation (SD) units and random variances in log SD units.

	**Estimate [95% CI]**
Fixed effects	
Intercept	0.08 [-0.05, 0.22]
sexMale	0.03 [-0.07, 0.13]
treatmentlow	0.01 [-0.15, 0.16]
treatmentsaline	0.10 [-0.05, 0.25]
treatmentinjured	0.05 [-0.10, 0.22]
treatmenthandled	0.02 [-0.14, 0.17]
ageYoung	0.03 [-0.07, 0.14]
**assay2**	**-0.31 [-0.38, -0.23]**
Residual variances	
ageOld:sexFemale:treatmenthigh	-0.04 [-0.18, 0.11]
ageYoung:sexFemale:treatmenthigh	-0.13 [-0.34, 0.08]
ageOld:sexMale:treatmenthigh	0.01 [-0.16, 0.18]
ageYoung:sexMale:treatmenthigh	-0.26 [-0.46, -0.01]
ageOld:sexFemale:treatmentlow	-0.06 [-0.25, 0.12]
ageYoung:sexFemale:treatmentlow	-0.49 [-0.69, -0.26]
ageOld:sexMale:treatmentlow	-0.06 [-0.24, 0.12]
ageYoung:sexMale:treatmentlow	-0.11 [-0.32, 0.09]
ageOld:sexFemale:treatmentsaline	0.01 [-0.17, 0.18]
ageYoung:sexFemale:treatmentsaline	-0.23 [-0.43, -0.01]
Random intercepts	
ageOld:sexMale:treatmentsaline	-0.18 [-0.35, -0.00]
ageYoung:sexMale:treatmentsaline	-0.01 [-0.21, 0.19]
ageOld:sexFemale:treatmentinjured	-0.33 [-0.53, -0.10]
ageYoung:sexFemale:treatmentinjured	-0.27 [-0.46, -0.05]
ageOld:sexMale:treatmentinjured	-0.08 [-0.26, 0.10]
ageYoung:sexMale:treatmentinjured	-0.14 [-0.34, 0.05]
ageOld:sexFemale:treatmenthandled	-0.09 [-0.28, 0.10]
ageYoung:sexFemale:treatmenthandled	-0.15 [-0.35, 0.05]
ageOld:sexMale:treatmenthandled	0.03 [-0.17, 0.21]
ageYoung:sexMale:treatmenthandled	-0.12 [-0.31, 0.06]
sexFemale:ageOld:treatmenthigh	0.16 [0.01, 0.47]
sexMale:ageOld:treatmenthigh	0.27 [0.01, 0.64]
sexFemale:ageYoung:treatmenthigh	0.52 [0.10, 0.82]
sexMale:ageYoung:treatmenthigh	0.60 [0.23, 0.87]
sexFemale:ageOld:treatmentlow	0.30 [0.02, 0.64]
sexMale:ageOld:treatmentlow	0.34 [0.02, 0.68]
sexFemale:ageYoung:treatmentlow	0.52 [0.28, 0.74]
sexMale:ageYoung:treatmentlow	0.44 [0.04, 0.77]
sexFemale:ageOld:treatmentsaline	0.37 [0.03, 0.74]
sexMale:ageOld:treatmentsaline	0.27 [0.01, 0.58]

sexFemale:ageYoung:treatmentsaline	0.47 [0.10, 0.75]
sexMale:ageYoung:treatmentsaline	0.51 [0.04, 0.84]
sexFemale:ageOld:treatmentinjured	0.68 [0.42, 0.94]
sexMale:ageOld:treatmentinjured	0.37 [0.02, 0.70]
sexFemale:ageYoung:treatmentinjured	0.66 [0.38, 0.92]
sexMale:ageYoung:treatmentinjured	0.36 [0.02, 0.68]
sexFemale:ageOld:treatmenthandled	0.39 [0.03, 0.71]
sexMale:ageOld:treatmenthandled	0.44 [0.04, 0.80]
sexFemale:ageYoung:treatmenthandled	0.49 [0.08, 0.78]
sexMale:ageYoung:treatmenthandled	0.35 [0.03, 0.66]
Num.Obs.	1864
R2	0.228
RMSE	0.81

Table S 4: Treatment contrasts (± 95% Credible Intervals) of among-individual variation (V_a) for activity (distance travelled, cm) and exploration (latency to visit all quadrats of arena,s) expressed by young (9-d old) and old (31-d old) adult female and male Tenebrio molitor beetles. 'Control' individuals experienced either handling, injury or saline injection (see main text for details), 'Low' individuals received a low dose of lipopolysacchardie (LPS), and 'High' individuals received a high dose of LPS.

	**Young**		**Old**	
**Contrast**	**Distance travelled**	**Quadrant visitation**	**Distance travelled**	**Quadrant visitation**
(a) Females				
Handled vs. Injured	-0.144 (-0.426, 0.160)	-0.193 (-0.591, 0.170)	-0.254 (-0.661, 0.185)	-0.305 (-0.656, 0.047)
Handled vs. Saline	-0.141 (-0.400, 0.125)	0.023 (-0.317, 0.352)	0.113 (-0.247, 0.456)	0.001 (-0.320, 0.329)
Handled vs. Low	-0.023 (-0.272, 0.243)	-0.019 (-0.332, 0.283)	0.015 (-0.398, 0.415)	0.049 (-0.241, 0.329)
Handled vs. High	-0.219 (-0.518, 0.093)	-0.028 (-0.385, 0.349)	0.186 (-0.125, 0.507)	0.120 (-0.091, 0.365)
Injured vs. Saline	0.002 (-0.311, 0.324)	0.216 (-0.152, 0.575)	0.367 (-0.029, 0.768)	0.307 (-0.039, 0.695)
Injured vs. Low	0.121 (-0.207, 0.419)	0.174 (-0.184, 0.505)	0.270 (-0.192, 0.710)	0.354 (0.002, 0.666)
Injured vs. High	-0.076 (-0.453, 0.264)	0.164 (-0.196, 0.584)	0.440 (0.062, 0.803)	0.426 (0.130, 0.711)
Saline vs. Low	0.119 (-0.161, 0.408)	-0.042 (-0.358, 0.233)	-0.097 (-0.484, 0.286)	0.047 (-0.267, 0.338)
Saline vs. High	-0.078 (-0.405, 0.256)	-0.051 (-0.387, 0.319)	0.073 (-0.211, 0.377)	0.119 (-0.106, 0.377)
Low vs. High	-0.197 (-0.539, 0.116)	-0.009 (-0.343, 0.318)	0.171 (-0.171, 0.537)	0.072 (-0.109, 0.298)
(b) Males				
Handled vs. Injured	-0.061 (-0.319, 0.189)	-0.008 (-0.291, 0.273)	0.199 (-0.160, 0.555)	0.059 (-0.305, 0.396)

Handled vs. Saline	-0.181 (-0.447, 0.058)	-0.130 (-0.469, 0.248)	-0.072 (-0.489, 0.340)	0.124 (-0.179, 0.439)
Handled vs. Low	-0.025 (-0.267, 0.192)	-0.073 (-0.405, 0.233)	0.226 (-0.140, 0.569)	0.077 (-0.263, 0.426)
Handled vs. High	-0.122 (-0.430, 0.173)	-0.231 (-0.553, 0.137)	0.055 (-0.323, 0.450)	0.112 (-0.214, 0.454)
Injured vs. Saline	-0.120 (-0.423, 0.175)	-0.122 (-0.493, 0.215)	-0.271 (-0.586, 0.089)	0.066 (-0.176, 0.339)
Injured vs. Low	0.036 (-0.250, 0.307)	-0.064 (-0.381, 0.269)	0.027 (-0.230, 0.295)	0.018 (-0.292, 0.298)
Injured vs. High	-0.061 (-0.404, 0.289)	-0.223 (-0.558, 0.123)	-0.144 (-0.452, 0.151)	0.054 (-0.219, 0.354)
Saline vs. Low	0.156 (-0.118, 0.425)	0.058 (-0.355, 0.424)	0.298 (-0.035, 0.627)	-0.048 (-0.299, 0.205)
Saline vs. High	0.059 (-0.285, 0.388)	-0.101 (-0.521, 0.303)	0.126 (-0.262, 0.479)	-0.012 (-0.243, 0.206)
Low vs. High	-0.097 (-0.415, 0.216)	-0.158 (-0.541, 0.212)	-0.171 (-0.469, 0.115)	0.036 (-0.229, 0.319)

Table S 5: Treatment contrasts (± 95% Credible Intervals) of within-individual (V_w) variation for activity (distance travelled, cm), exploration (latency to visit all quadrats of arena,s), and boldness (time in centre of arena, s) expressed by young (9-d old) and old (31-d old) adult female and male Tenebrio molitor beetles. 'Control' individuals experienced either handling, injury or saline injection (see main text for details), 'Low' individuals received a low dose of lipopolysacchardie (LPS), and 'High' individuals received a high dose of LPS. Fixed effect estimates that differ from zero are in bold text.

	**Young**		**Old**	
**Contrast**	**Distance travelled**	**Quadrant visitation**	**Distance travelled**	**Quadrant visitation**
(a) Females				
Handled vs. Injured	-0.110 (-0.464, 0.234)	0.157 (-0.235, 0.587)	0.097 (-0.315, 0.547)	0.318 (-0.063, 0.780)
Handled vs. Saline	0.099 (-0.206, 0.389)	0.103 (-0.314, 0.545)	-0.207 (-0.670, 0.254)	-0.175 (-0.658, 0.326)
Handled vs. Low	-0.062 (-0.407, 0.253)	0.363 (-0.001, 0.697)	-0.282 (-0.810, 0.244)	-0.047 (-0.513, 0.398)
Handled vs. High	-0.072 (-0.433, 0.256)	-0.031 (-0.506, 0.414)	**-0.494 (-1.000, 0.001)**	-0.088 (-0.489, 0.372)
Injured vs. Saline	0.209 (-0.127, 0.555)	-0.053 (-0.449, 0.334)	-0.304 (-0.749, 0.148)	**-0.493 (-0.931, -0.060)**
Injured vs. Low	0.048 (-0.316, 0.427)	0.206 (-0.087, 0.531)	-0.379 (-0.892, 0.123)	-0.364 (-0.783, 0.041)
Injured vs. High	0.038 (-0.352, 0.431)	-0.188 (-0.648, 0.207)	**-0.592 (-1.071, -0.116)**	**-0.406 (-0.764, -0.016)**
Saline vs. Low	-0.161 (-0.487, 0.148)	0.260 (-0.077, 0.590)	-0.075 (-0.618, 0.465)	0.129 (-0.378, 0.639)
Saline vs. High	-0.171 (-0.520, 0.146)	-0.134 (-0.596, 0.285)	-0.288 (-0.794, 0.241)	0.087 (-0.351, 0.541)
Low vs. High	-0.010 (-0.371, 0.365)	-0.394 (-0.777, -0.015)	-0.213 (-0.786, 0.359)	-0.042 (-0.475, 0.383)
(b) Males				
Handled vs. Injured	-0.118 (-0.527, 0.292)	0.024 (-0.367, 0.460)	0.058 (-0.404, 0.569)	0.198 (-0.300, 0.744)

Handled vs. Saline	0.197 (-0.136, 0.551)	-0.207 (-0.728, 0.269)	0.293 (-0.153, 0.780)	0.351 (-0.110, 0.837)
Handled vs. Low	-0.081 (-0.467, 0.307)	-0.027 (-0.513, 0.393)	-0.082 (-0.573, 0.438)	0.157 (-0.350, 0.682)
Handled vs. High	-0.236 (-0.713, 0.222)	0.176 (-0.247, 0.586)	0.271 (-0.162, 0.763)	0.040 (-0.481, 0.566)
Injured vs. Saline	0.315 (-0.075, 0.717)	-0.231 (-0.728, 0.251)	0.235 (-0.167, 0.641)	0.153 (-0.263, 0.549)
Injured vs. Low	0.038 (-0.394, 0.479)	-0.052 (-0.500, 0.398)	-0.141 (-0.596, 0.317)	-0.041 (-0.496, 0.437)
Injured vs. High	-0.117 (-0.633, 0.379)	0.151 (-0.277, 0.589)	0.212 (-0.194, 0.628)	-0.159 (-0.630, 0.299)
Saline vs. Low	-0.278 (-0.652, 0.091)	0.180 (-0.363, 0.695)	-0.376 (-0.809, 0.056)	-0.194 (-0.610, 0.222)
Saline vs. High	-0.432 (-0.900, -0.005)	0.383 (-0.147, 0.841)	-0.023 (-0.417, 0.357)	-0.311 (-0.723, 0.115)
Low vs. High	-0.155 (-0.635, 0.338)	0.203 (-0.216, 0.696)	0.353 (-0.074, 0.786)	-0.117 (-0.607, 0.336)
